# Automated evaluation of autoantibodies on human epithelial-2 cells as an approach to standardize cell-based immunofluorescence tests

**DOI:** 10.1186/ar2949

**Published:** 2010-03-09

**Authors:** Karl Egerer, Dirk Roggenbuck, Rico Hiemann, Max-Georg Weyer, Thomas Büttner, Boris Radau, Rosemarie Krause, Barbara Lehmann, Eugen Feist, Gerd-Rüdiger Burmester

**Affiliations:** 1 Department of Rheumatology and Clinical Immunology, Charité-Universitätsmedizin Berlin, Charitéplatz 1, 10117 Berlin, Germany; 2 GA Generic Assays GmbH, Ludwig-Erhard-Ring 3, 15287 Dahlewitz/Berlin, Germany; 3 University of Applied Science Lausitz, Großenhainer Str. 57, 01968 Senftenberg, Germany; 4 Medizinisches Versorgungszentrum für Laboratoriumsmedizin, Mikrobiologie, Virologie und Infektionsepidemiologie, Hygiene und Umweltmedizin, Dr. Löer - Dr. Treder und Kollegen, Hafenweg 11, 48155 Münster, Germany

## Abstract

**Introduction:**

Analysis of autoantibodies (AAB) by indirect immunofluorescence (IIF) is a basic tool for the serological diagnosis of systemic rheumatic disorders. Automation of autoantibody IIF reading including pattern recognition may improve intra- and inter-laboratory variability and meet the demand for cost-effective assessment of large numbers of samples. Comparing automated and visual interpretation, the usefulness for routine laboratory diagnostics was investigated.

**Methods:**

Autoantibody detection by IIF on human epithelial-2 (HEp-2) cells was conducted in a total of 1222 consecutive sera of patients with suspected systemic rheumatic diseases from a university routine laboratory (n = 924) and a private referral laboratory (n = 298). IIF results from routine diagnostics were compared with a novel automated interpretation system.

**Results:**

Both diagnostic procedures showed a very good agreement in detecting AAB (kappa = 0.828) and differentiating respective immunofluorescence patterns. Only 98 (8.0%) of 1222 sera demonstrated discrepant results in the differentiation of positive from negative samples. The contingency coefficients of chi-square statistics were 0.646 for the university laboratory cohort with an agreement of 93.0% and 0.695 for the private laboratory cohort with an agreement of 90.6%, *P *< 0.0001, respectively. Comparing immunofluorescence patterns, 111 (15.3%) sera yielded differing results.

**Conclusions:**

Automated assessment of AAB by IIF on HEp-2 cells using an automated interpretation system is a reliable and robust method for positive/negative differentiation. Employing novel mathematical algorithms, automated interpretation provides reproducible detection of specific immunofluorescence patterns on HEp-2 cells. Automated interpretation can reduce drawbacks of IIF for AAB detection in routine diagnostics providing more reliable data for clinicians.

## Introduction

Disease-specific autoantibodies (ABBs) are a serological phenomenon of systemic rheumatic conditions and autoimmune liver disorders. Despite the development of enzyme-linked immunosorbent immunoassay (ELISA) and multiplexing technologies for the detection of disease-specific AABs, the screening for anti-nuclear antibodies (ANAs) by indirect immunofluorescence (IIF) assays remains a standard method in the current diagnostic approach [1-6]. Several substrates have been proposed for ANA IIF assays; however, the screening for non-organ-specific AABs on human epithelial (HEp-2) cells is the most established method used [7-11]. In general, assessment of ANAs is followed by detection of specific AABs to, for example, extractable nuclear antigens (ENAs) and cytoplasmic antigens by immunoassays employing purified native or recombinant antigens. This two-stage approach comprises the following benefits: (a) highly sensitive screening of the most frequent and clinically relevant non-organ-specific AABs, (b) optimal combination with other assay techniques for the downstream differentiation of AAB reactivities based on the IIF pattern detected and the diagnosis suspected, (c) assessment of clinically relevant AABs without the need for further testing (for example, anti-centromere AABs), and (d) evaluation of AABs detectable only by IIF in case of unknown autoantigenic targets or non-available commercial assays [12-14]. Due to the key position of ANA screening in the serological diagnosis of systemic rheumatic diseases, consistent reproducibility and high quality of HEp-2 cell-based IIF assays are required [8,15,16]. However, the visual and therefore subjective evaluation of cell-based IIF assays complicates the standardized and reproducible evaluation of HEp-2 cell assays. Interpretation of immunofluorescence patterns is influenced by the knowledge and individual qualification of the investigator. Thus, a high intra- and interlaboratory variability is common and represents a major diagnostic problem, especially in non-specialized laboratories [17,18]. Automated reading of immunofluorescence patterns by automated interpretation systems with intelligent pattern recognition can overcome this issue [18,19]. In addition, automation of IIF pattern reading can provide a reliable basis for cost-effective serological diagnostics for laboratories with large sample numbers. In particular, the opportunity of modern electronic data management alleviates the heavy workload in such laboratories.

In this study, we compared the first automated interpretation system available for cell-based IIF with the currently established visual evaluation method in routine diagnostics of both a university and a private rheumatology referral laboratory. Visual findings of positive/negative discrimination and AAB pattern detection were compared with data automatically obtained by this system. Perspectives of automated interpretation of cell-based IIF tests will be discussed.

## Materials and methods

Consecutive serum samples of 924 patients with a suspected diagnosis of systemic rheumatic diseases were referred to the routine laboratory at the Department of Rheumatology and Clinical Immunology of the Charité Universitätsmedizin Berlin. ANAs were determined using a HEp-2 cell-based assay. Samples with a titer of 1 in 320 or higher were scored as positive and subsequently tested for AABs against ENA. Samples with a titer of 1 in 80 or 1 in 160 were scored as weakly positive. Moreover, to assess the performance of the automated interpretation in a different setting, 288 consecutive serum samples were tested from a private referral laboratory. This laboratory receives mainly samples from general practitioners and small- and medium-sized hospitals to provide serological findings for the clarification of suspected rheumatic symptoms. Final diagnoses are usually not reported to the laboratory. The study was approved by the local ethics committee (EA1/001/06). Written informed consent was obtained from each patient.

### Detection of anti-nuclear antibodies by HEp-2 cell assay

ANAs in patient samples were assessed by commercial ANA assays in accordance with the instructions of the manufacturer (GA Generic Assays GmbH, Dahlewitz, Germany). Briefly, samples diluted in phosphate-buffered saline were incubated on HEp-2 cells fixed on glass slides in a moisture chamber for 30 minutes at room temperature (RT). The screening dilution was 1 in 160, except for individuals younger than 14 years old, in whom a screening dilution of 1 in 80 was applied. After washing, bound AABs were detected by incubation with fluorescein isothiocyanate-conjugated sheep anti-human immunoglobulin for 30 minutes at RT. Subsequently, slides were washed, embedded with 4',6-diamidino-2-phenylindol (DAPI)-containing medium, and assessed either visually with a fluorescence microscope (Axiovert 40; Carl Zeiss, Jena, Germany, and Eurostar; Euroimmun AG, Lübeck, Germany) or automatically with the interpretation system (AKLIDES^®^; Medipan GmbH, Dahlewitz, Germany). Observers conducting the visual assessment were DR, M-GW, TB, RK, and BL.

### Automated interpretation of HEp-2 cell assay data

**Figure 1:**
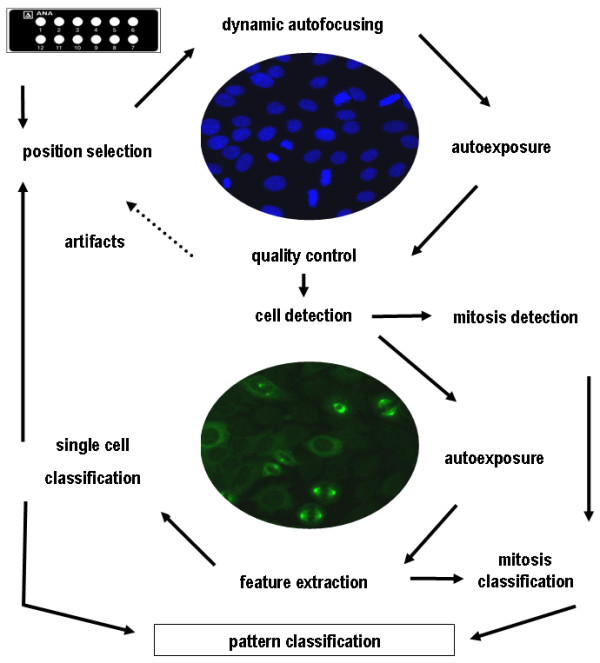
**Flowchart of automated human epithelial (HEp-2) cell assay interpretation by the automated reading system **[18]. The fundamental analysis chain of the image processing by the automated system is divided into acquisition, quality control, segmenting, object description, and object classification. Segmented objects were described by boundary, regional, topological, and texture/surface descriptors. Digital features were combined to rules, analogous to rules defined by experts.

The concept of the automated interpretation system AKLIDES^® ^for evaluation of ANAs including pattern recognition is based on IIF using HEp-2 cells (Figure 1) [18,19]. Briefly, IIF patterns of serum samples were assessed automatically on HEp-2 cells (GA Generic Assays GmbH) by using a motorized inverse microscope (Olympus IX81; Olympus Corporation, Tokyo, Japan) with a motorized scanning stage (Märzhäuser Wetzlar GmbH & Co. KG, Wetzlar, Germany), 400-nm and 490-nm light-emitting diodes (CoolLED Ltd., Andover, UK), and a grey-scale camera (Kappa, Gleichen, Germany). The interpretation system is controlled by the specially designed software (AKLIDES^®^), which consists of modules for device and autofocus control, image analysis, and pattern recognition algorithms. The novel autofocus based on Haralick's image characterization of objects through grey-scale transition used DAPI as fluorescent dye for object recognition and focusing. To eliminate artifacts, an additional qualitative image analysis was performed by dividing the image into subobjects of equal size.

Object segmentation was conducted by histogram-based threshold algorithm followed by watershed transformation [20]. Segmented objects were characterized by regional, topological, and texture/surface descriptors. More than 1,400 object-describing criteria were implemented. Mitotic cells were identified by DAPI staining. Classification was achieved through the combination of structure and texture characteristics by definition of rules for each pattern.

Immunofluorescence image data were evaluated according to the following hierarchy: (a) positivity, (b) localization of staining (nuclear, cytoplasmic, chromatin of mitotic cells), and (c) determination of nuclear patterns: homogeneous (homogeneous or speckled pattern with specific staining of the metaphase chromatin), speckled (fine, medium, or coarse speckled staining of interphase nuclei), nucleolar (homogeneous or speckled staining of nucleoli with weak nuclear staining or without nuclear staining), centromere (more than 30 nuclear dots in the interphase nucleus and metaphase chromatin), and multiple nuclear dots (multiple dots, fewer than 30 nuclear dots in the interphase nucleus).

A reactivity index (RI) was calculated by combining absolute image intensity, contrast, and number of grey-scale levels of the total image for the assessment of image data. Since RI is influenced by exposure time, which depends on the highest image signal after exclusion of artifacts, even patterns with weak absolute signals like centrioles or nuclear dots can be detected. The determination of threshold values for the differentiation of positive signals was conducted on the basis of RI values of 200 normal blood donors.

With this software, the following six main patterns can be detected readily on HEp-2 cells: cytoplasmic, homogeneous, speckled, nucleolar, centromere, and multiple nuclear dots. Further stratification of nuclear or cytoplasmic patterns was performed by retrospective visual examination if required for discussion of differing results. Given an average workload of about 50 determinations a day, the system provides sufficient data storage capacity for 1 year.

### Statistical analysis

Chi-square test was used to check the relationship between the two classification systems. To test for the strength of agreement, inter-rater agreement statistics was conducted [21]. McNemar test was performed to check the difference for paired proportions. *P *values of less than 0.05 were considered significant. Calculations were performed by using MedCalc statistical software (MedCalc, Mariakerke, Belgium).

## Results

### Comparison of positive and negative findings of patient sera referred to a routine university laboratory

**Figure 2:**
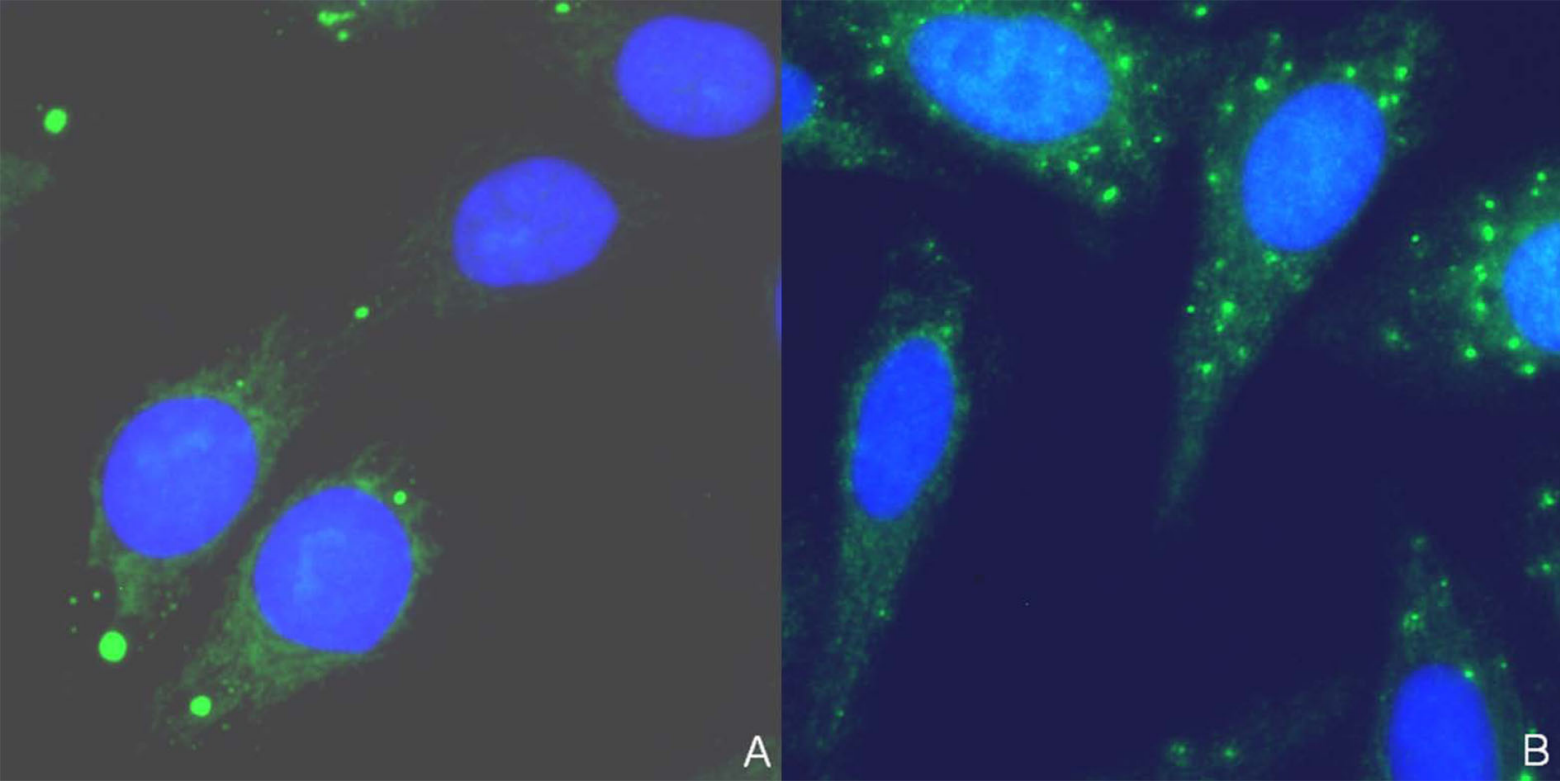
**Immunofluorescence patterns of two sera (a, b) which were both scored as negative by visual examination but demonstrated positive cytoplasmic staining by AKLIDES^® ^system**. Green color: fluorescein isothiocyanate staining of autoantibody; blue color: 4',6-diamidino-2-phenylindol staining of chromatin.

Consecutive sera of 924 patients with suspected systemic rheumatic disease were evaluated for the presence of ANAs in a routine university laboratory. ANAs were detected by HEp-2 cell assay and interpreted either visually by an experienced investigator or by automated reading and pattern recognition with the system. Samples were blinded for evaluation. Automated evaluation reported 546 sera (59.1%) as positive, 140 sera (15.1%) as weakly positive, and 238 sera (25.8%) as negative in regard to the presence of ANAs. Out of the 546 positively scored sera, 543 sera (99.5%) were confirmed by visual examination as positive or weakly positive (Table 1). Two of the three discrepant sera demonstrated a cytoplasmic pattern, which was assessed as a negative ANA by visual examination (Figure 2). Cytoplasmic patterns of these samples were defined by retrospective examination. The third discrepant sample showed an artifact, leading to a positive finding by the automated system.

**Table 1:** Comparison of automated and visual anti-nuclear antibody interpretation in a university routine laboratory

		Visual interpretation			
		
		Positive	Weak positive	Negative	Number (percentage)
Automated interpretation	Positive	139	404	3	546 (59.1%)
	Weak positive	1	113	26	140 (15.1%)
	Negative	0	39	199	238 (25.8%)
	Number (percentage)	140 (15.1%)	556 (60.2%)	228 (24.7)	924

Out of 140 sera scored as weakly positive by the system, 113 sera (80.7%) were also interpreted as weakly positive by visual examination and one serum (1.0%) was interpreted as positive by visual examination. The 26 sera assessed as negative by visual examination (18.6%) demonstrated mainly weakly positive speckled staining of the nucleus in the automated system and this was scored as irrelevant by visual reading.

Out of 238 sera scored negative by the system, 199 sera (83.6%) were also assessed as negative by visual examination. In fact, none of these negative samples was evaluated as positive by visual examination. Only 39 sera (16.4%) were assessed as weakly positive, showing a titer of 1 in 80 with unspecific patterns by visual assessment. Thus, there was an agreement of 93.0% (859/924) regarding the discrimination of positive and negative samples by both approaches in this university laboratory. Chi-square statistics revealed a contingency coefficient of 0.646 (*P *< 0.0001). When weakly positive and definitely positive samples were combined into one group, the difference of 1.08% according to the McNemar test between both methods for positive/negative differentiation was not significant (95% confidence interval [CI] -0.77% to 2.84%; *P *= 0.25).

### Comparison of pattern assessment of patient sera referred to a routine university laboratory

There was a high agreement of 90.1% (492/546) comparing the visually and automatically defined fluorescence patterns of the samples reported positive by the automated system. The differing samples mainly demonstrated mixed patterns, which were assessed by visual expert examination and automated reading algorithms of the automated system with different emphasis of one or the other underlying pattern. Investigators emphasized the staining of nucleoli when assessing the combination of speckled and nucleolar patterns visually. In contrast, the mathematical software algorithms included the denser distribution of the speckled pattern with more value into decision making. A similar situation was found with the combination of nuclear and cytoplasmic patterns. When this mixed pattern was assessed, visual interpretation of experts tended to emphasize the nuclear staining (speckled, nucleolar). In contrast, the system algorithms emphasize the cytoplasmic staining in case of high-fluorescence signals.

**Figure 3:**
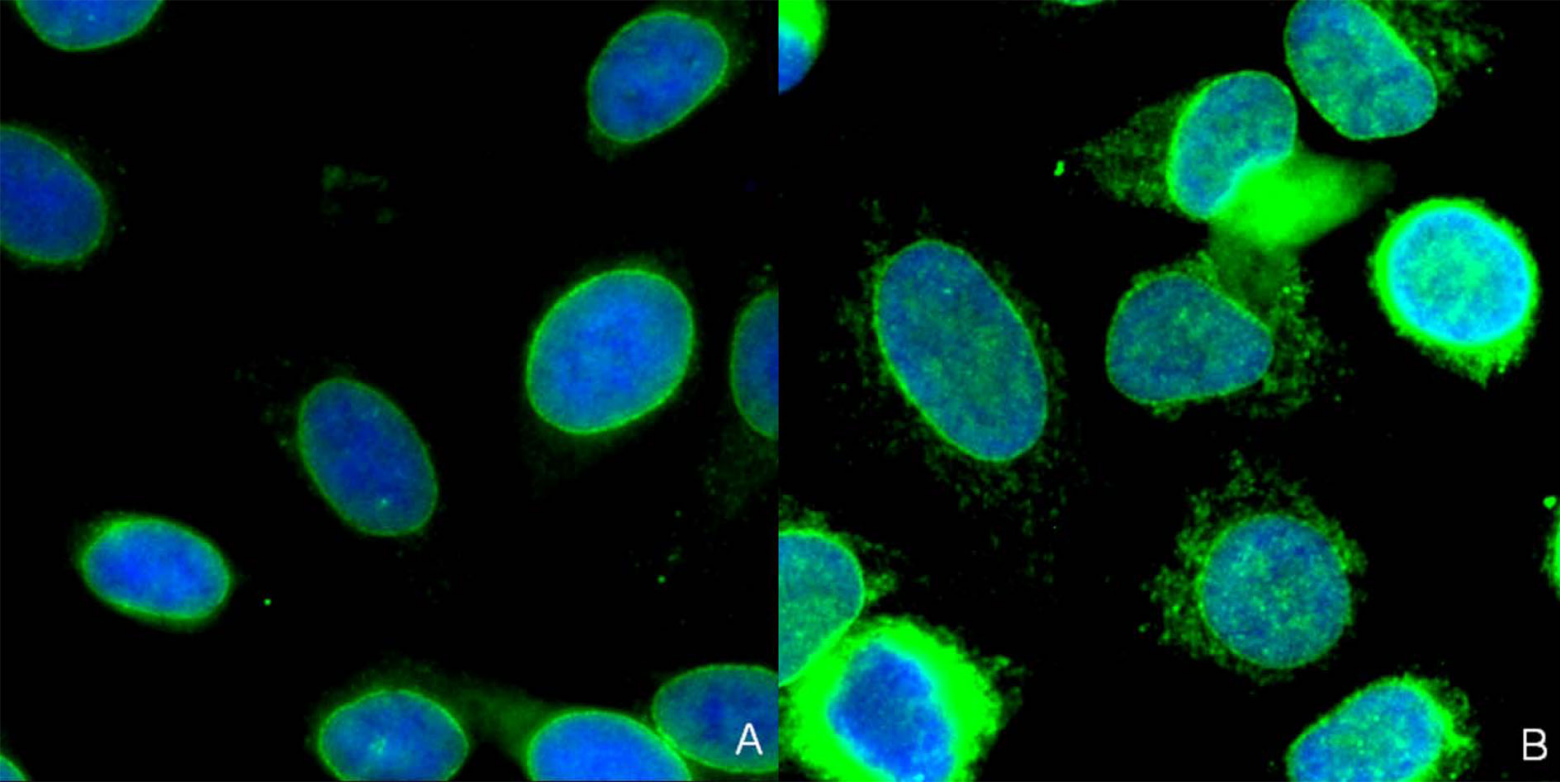
**Immunofluorescence patterns of two sera (a, b) which were both assessed as positive with speckled pattern by AKLIDES^® ^system but revealed staining of the nuclear membrane by visual examination**. Green color: fluorescein isothiocyanate staining of autoantibody; blue color: 4',6-diamidino-2-phenylindol staining of chromatin.

**Figure 4:**
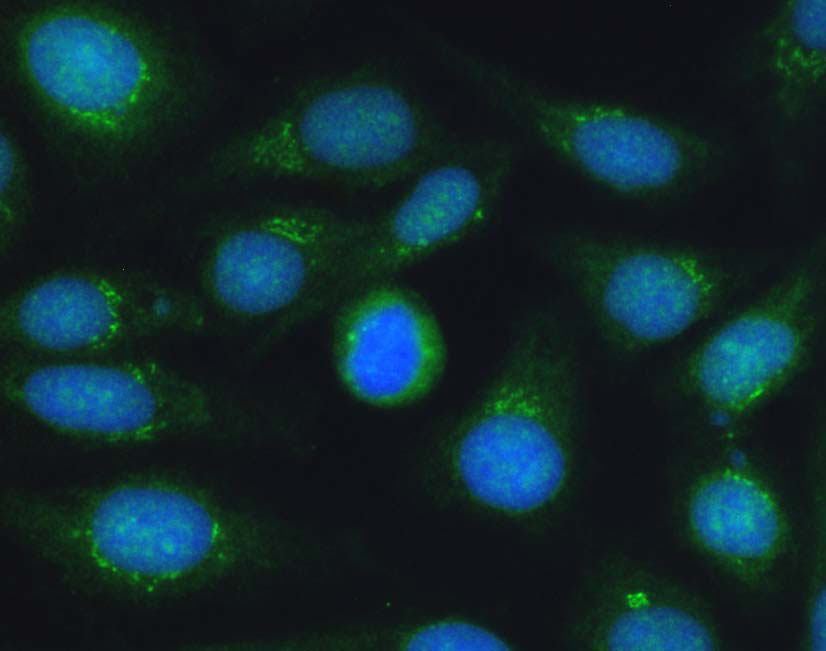
**Immunofluorescence pattern with staining of the Golgi complex, which was identified by AKLIDES^® ^system as cytoplasmic speckled pattern**. Green color: fluorescein isothiocyanate staining of autoantibody; blue color: 4',6-diamidino-2-phenylindol staining of chromatin.

Discrepant assessment of patterns was found with sera containing antibodies to nuclear membrane targets. These patterns were evaluated by the system algorithms as speckled. In contrast, the visual assessment clearly detected the increased speckled staining at the border of the nucleus (Figure 3). Sera containing antibodies to the Golgi complex were assessed as weakly speckled nuclear pattern by the software algorithms (Figure 4).

The pattern comparison of the 140 samples scored as weakly positive by the automated system demonstrated an agreement of 74.3% (104 sera). Discrepant samples again showed weak speckled nuclear staining. In summary, comparison of fluorescence pattern recognition of the 686 positive and weakly positive findings by the system with visual examination demonstrated an agreement in 596 sera (86.9%).

### Comparison of positive and negative findings of patient sera referred to a private laboratory

Furthermore, 298 consecutive sera referred to a private laboratory for the detection of ANAs were compared with ANA assessment by the system after routine visual evaluation by an expert (Table 2). Samples were blinded for evaluation. Automated interpretation with the system scored 57 sera (19.1%) of these 298 sera as positive, 16 (5.4%) as weakly positive, and 225 (75.5%) as negative. Of the 57 samples assessed as positive by the system, 55 sera (96.5%) were assessed as positive or weakly positive by visual evaluation.

**Table 2:** Comparison of automated and visual anti-nuclear antibody interpretation in a referral routine laboratory

		Visual interpretation			
		
		Positive	Weak positive	Negative	Number (percentage)
Automated interpretation	Positive	44	11	2	57 (19.1%)
	Weak positive	0	12	4	16 (5.4%)
	Negative	0	24	201	225 (75.5%)
	Number (percentage)	44 (14.8%)	47 (15.8%)	207 (69.4%)	298

Evaluation by automated interpretation scored 16 sera as weakly positive. Visual assessment determined 12 (75.0%) of these 16 sera to be positive with the same fluorescence pattern (100.0%). The four sera (25.0%) scored as negative demonstrated weak speckled fluorescence patterns in the system.

Out of 225 sera assessed as negative by the automated system, 201 sera (89.3%) were confirmed as negative by visual examination. The 24 discrepant sera (10.7%) that were scored as weakly positive with speckled or nucleolar patterns by the investigator and did not reach the threshold level in the automated system demonstrated no antibodies to ENA by other techniques. Thus, agreement in this patient cohort regarding the differentiation of positive and negative samples was 90.6% (270/298). Chi-square statistics revealed a contingency coefficient of 0.695 (*P *< 0.0001).

There was a significant difference of 6.04% (95% CI 2.30% to 8.50%; *P *= 0.0019) for both methods in this patient cohort by combining positive and weakly positive samples. When weakly positive results were excluded and positive and negative samples only were taken into account, the difference of 0.81% (95% CI -0.50% to 0.81%) was not significant.

**Figure 5:**
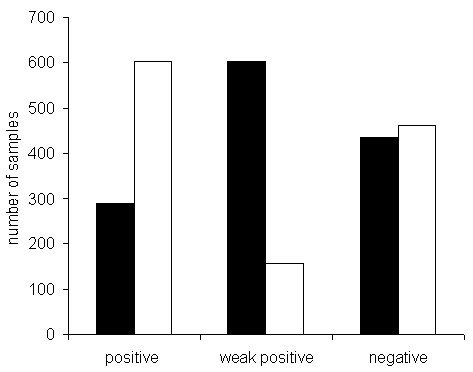
**Comparison of positive and negative findings of 1,222 patient sera referred to a routine university laboratory (white bars) and a private laboratory (black bars)**. Negative samples demonstrated titers below 1 in 80, weak positive samples 1 in 80 or 1 in 160, and positive samples 1 in 320 or above.

In total, only 98 out of 1,222 sera (8.0%) demonstrated discrepant results regarding positive and negative differentiation by visual and automated interpretation (Figure 5). When positive and weakly positive samples were combined into one group, the strength of agreement was very good (kappa = 0.828, 95% CI 0.795 to 0.860). For the assessment of one sample, the automated system required 60 seconds on average in a walk-away mode.

### Comparison of pattern assessment of patient sera referred to a private laboratory

Fifty-one of the 55 sera (92.7%) of sera scored positive by the automated system showed agreement in fluorescence pattern detection by visual and automated interpretation. Discrepant results were obtained when the AKLIDES^® ^software algorithms assessed cytoplasmic fluorescence signals as nuclear staining due to the superposition of the nucleus by the cytoplasmic staining.

## Discussion

The detection of AABs like ANAs by IIF was one of the first techniques available in routine laboratories for the serological diagnosis of systemic rheumatic diseases [22,23]. ANAs were even included in the classification criteria of systemic lupus erythematosus [24]. However, due to insufficient automation, poor standardization, and need of extensive expert experience in pattern recognition, automated ELISA and recently multiplexing assays have frequently been used for ANA assessment [25,26]. Not only for ANAs, there is an ongoing debate whether these new techniques may replace immunofluorescence given that their limited sensitivity might be problematic for a screening method [27-31].

Until recently, reliable diagnostic tools for the automated interpretation of cell-based IIF tests like ANA detection on HEp-2 cells have not been available for routine laboratories. However, the use of digital images of HEp-2 cell-based assays for diagnostic aims [32,33] and the superiority of automated in contrast to subjective pattern classification have already been demonstrated [34]. Thus, the objective of this study was the comparison of the current visual subjective interpretation of HEp-2 cell-based assays for ANA detection with results obtained by the first automated interpretation system. Since the detection of ANAs is employed as serological screening for patients with suspected rheumatic disorders on the one hand and is part of classification criteria of systemic rheumatic diseases on the other, two patient groups tested with differing laboratory background regarding experience in ANA detection and prevalence of disease were included in the study. Consecutive sera of both a university laboratory specialized in rheumatic disease diagnostics and a private referral laboratory covering hospitals and out-patient departments were included in the study.

The basic precondition for the use of automated interpretation systems in routine diagnostics is the correct and reproducible differentiation of positive and negative samples. The comparison of visually and automatically obtained findings is hindered due to the lack of readily available standards with defined cutoffs for the definition of positive signals on HEp-2 cells in IIF assays. The Centers for Disease Control and Prevention (Atlanta, GA, USA) provide serum standards for specific patterns which are recommended to be employed for quality management. Laboratories providing ANA detection by HEp-2 cell assays frequently report different titers since cutoffs depend on technical equipment, expert knowledge, and patient population of the corresponding laboratory.

By means of the automated system, a very good agreement of 92.0% (kappa = 0.828) was obtained for the differentiation of positive and negative samples comparing automated interpretation with visual assessment by experienced examiners in different patient cohorts.

There was no significant difference for either interpretation method for the university patient cohort in differentiating positive from negative samples in our study. After exclusion of the weakly positive samples, the difference for both interpretation methods was also not significant for the patient cohort evaluated in the private referral laboratory. In such a cohort, a low prevalence of systemic rheumatic disease is usually expected. Samples with low ANA titers of 1 in 160 or less are not suggested to be subjected to further anti-ENA testing unless systemic rheumatic disease is strongly suspected [35]. In this context, automated interpretation of ANAs of this study is not significantly different from visual reading by experts regarding at least samples with ANA titers of more than 1 in 160.

The relatively high variability of routinely employed pattern recognition of ANA fluorescence images on HEp-2 cells is a challenge for the implementation of automated pattern recognition. Thus, different criteria exist, for example, for the description of coarse and fine speckled patterns [36]. Otherwise, a nucleolar pattern is usually defined by the positive staining of nucleoli but has to be specified by further staining of the chromatin region. The nucleolar staining can appear as homogeneous, clumpy, fine speckled, and speckled with mitotic dots and can be associated with AABs against PM-Scl complex, TH/To, fibrillarin, RNA polymerase I, and RNA helicase II. Anti-polymerase III or Ku AABs often demonstrate a fine speckled staining of the interphase chromatin additionally. Initiatives for the standardization of fluorescence patterns on HEp-2 cells for ANA detection have aimed at bridging the gap between routine diagnostics and science. Thus, five main patterns are recommended for the differentiation of nuclear staining patterns [17]. Elementary evaluation models for single patterns regarding the classification of pleomorphic patterns have already been developed [33].

The drawbacks of recently published approaches for automated pattern recognition appear to include an over-evaluation of final steps in image assessment like object extraction and classification [37-40]. In particular, self-learning classificators [39] have to be reviewed critically since local erroneous self-learning cannot lead to improvement of interlaboratory variability. Frequently, highly qualitative images are preselected, paving the way for human bias of subsequent findings.

In our study, agreement of pattern recognition between automated and visual assessment was 85.0%. This congruence reached 90.0% when only positive samples were taken into account. Weakly positive samples detected by visual examination demonstrated titers below 1 in 160. The latter finding confirms data of a recently published study [35].

The high agreement of our study between automated and visual interpretation of AABs results supports recent data showing that the success of automated interpretation systems depends essentially on the first processing steps like qualitative image acquisition and quality control of object identification [18,38].

The system used in the present study with novel pattern recognition algorithms for the automated assessment of HEp-2 cell assays may be employed for efficient AAB screening, especially in laboratories with high numbers of determination due to cost-effective management of data and human resources. The system can be readily implemented into routine diagnostics with reasonable demand of operator training. Findings provided by the system should be approved by an expert with experience in routine ANA reading due to the difficulty in assessing sera with differing AABs resulting in mixed patterns. Titer prediction enabled by the standardization of the fluorescence signal can further improve cost-efficiency [19,41].

## Conclusions

The standardized evaluation of HEp-2 cell assays by automated interpretation systems can pave the way for reproducible and comparable results in and between laboratories. Archiving of digitized image data improves data management and provides the basis for efficient exchange of data. Automated interpretation systems for cell-based IIF assays can minimize the drawbacks regarding other automated techniques and strengthen the role of immunofluorescence for serological screening of autoimmune diseases.

## Abbreviations

AAB: autoantibody; ANA: anti-nuclear antibody; CI: confidence interval; DAPI: 4',6-diamidino-2-phenylindol; ELISA: enzyme-linked immunosorbent immunoassay; ENA: extractable nuclear antigen; HEp-2: human epithelial; IIF: indirect immunofluorescence; RI: reactivity index; RT: room temperature.

## Competing interests

DR is a shareholder of GA Generic Assays GmbH and Medipan GmbH. Both companies are diagnostic manufacturers. The other authors declare that they have no competing interests.

## Authors' contributions

KE, DR, RH, TB, BR, RK, and BL carried out the immunofluorescence assays manually and automatically. EF, MGW and GRB conceived of the study and participated in its design and coordination and helped to draft the manuscript. All authors read and approved the final manuscript.

## References

[B1] Damoiseaux JG Tervaert JW From ANA to ENA: How to proceed? Autoimmun Rev 2006 5 10 17 10.1016/j.autrev.2005.05.007 16338206

[B2] Fritzler MJ Challenges to the use of autoantibodies as predictors of disease onset, diagnosis and outcomes Autoimmun Rev 2008 7 616 620 10.1016/j.autrev.2008.06.007 18603023

[B3] Ardoin SP Pisetsky DS Developments in the scientific understanding of lupus Arthritis Res Ther 2008 10 218 10.1186/ar2488 18947369 PMC2592776

[B4] Peene I Meheus L Veys EM De Keyser F Detection and identification of antinuclear antibodies (ANA) in a large and consecutive cohort of serum samples referred for ANA testing Ann Rheum Dis 2001 60 1131 1136 10.1136/ard.60.12.1131 11709455 PMC1753451

[B5] Sordet C Goetz J Sibilia J Contribution of autoantibodies to the diagnosis and nosology of inflammatory muscle disease Joint Bone Spine 2006 73 646 654 10.1016/j.jbspin.2006.04.005 17110150

[B6] Worman HJ Courvalin JC Antinuclear antibodies specific for primary biliary cirrhosis Autoimmun Rev 2003 2 211 217 10.1016/S1568-9972(03)00013-2 12848948

[B7] Hartung K Seelig HP Laboratory diagnostics of systemic autoimmune diseases. Part 1. Collagenoses Z Rheumatol 2006 65 709 724 10.1007/s00393-006-0125-5 17119898

[B8] Tozzoli R Bizzaro N Tonutti E Villalta D Bassetti D Manoni F Piazza A Pradella M Rizzotti P Italian Society of Laboratory Medicine Study Group on the Diagnosis of Autoimmune Diseases Guidelines for the laboratory use of autoantibody tests in the diagnosis and monitoring of autoimmune rheumatic diseases Am J Clin Pathol 2002 117 316 324 10.1309/Y5VF-C3DM-L8XV-U053 11863229

[B9] Wiik AS Gordon TP Kavanaugh AF Lahita RG Reeves W van Venrooij WJ Wilson MR Fritzler M IUIS/WHO/AF/CDC Committee for the Standardization of Autoantibodies in Rheumatic and Related Diseases Cutting edge diagnostics in rheumatology: the role of patients, clinicians, and laboratory scientists in optimizing the use of autoimmune serology Arthritis Rheum 2004 51 291 298 10.1002/art.20229 15077275

[B10] Wiik AS Anti-nuclear autoantibodies: clinical utility for diagnosis, prognosis, monitoring, and planning of treatment strategy in systemic immunoinflammatory diseases Scand J Rheumatol 2005 34 260 268 10.1080/03009740500202664 16195158

[B11] Craig WY Ledue TB Johnson AM Ritchie RF The distribution of antinuclear antibody titers in "normal" children and adults J Rheumatol 1999 26 914 919 10229416

[B12] Andrade LE Chan EK Peebles CL Tan EM Two major autoantigen-antibody systems of the mitotic spindle apparatus Arthritis Rheum 1996 39 1643 1653 10.1002/art.1780391006 8843854

[B13] Herrera-Esparza R Avalos-Diaz E Barbosa-Cisneros O Anti-NuMA antibodies: an uncommon specificity in scleroderma sera Rev Rhum Engl Ed 1999 66 315 318 10418059

[B14] Montecucco C Caporali R Cobianchi F Biamonti G Identification of autoantibodies to the I protein of the heterogeneous nuclear ribonucleoprotein complex in patients with systemic sclerosis Arthritis Rheum 1996 39 1669 1676 10.1002/art.1780391009 8843857

[B15] Brand F Martin F Philipp S Rößler J Hansen B Anderer U Difference in fluorescence pattern of cytoplasmic and nuclear antigens in cultivated human cells dependent on the applied fixation procedure Cell Prolif 2005 38 205

[B16] Du L Fukushima S Sallmyr A Manthorpe R Bredberg A Exposure of HEp-2 cells to stress conditions influences antinuclear antibody reactivity Clin Diagn Lab Immunol 2002 9 287 294 11874865 10.1128/CDLI.9.2.287-294.2002 PMC119933

[B17] Sack U Conrad K Csernok E Frank I Hiepe F Krieger T Kromminga A von Landenberg P Messer G Witte T Mierau R for the German EASI (European Autoimmunity Standardization Initiative) Autoantibody detection using indirect immunofluorescence on HEp-2 cells Ann N Y Acad Sci 2009 1173 166 173 10.1111/j.1749-6632.2009.04735.x 19758146

[B18] Hiemann R Büttner T Krieger T Roggenbuck D Sack U Conrad K Challenges of automated screening and differentiation of non-organ specific autoantibodies on HEp-2 cells Autoimmun Rev 2009 9 17 22 10.1016/j.autrev.2009.02.033 19245860

[B19] Hiemann R Hilger N Michel J Nitschke J Böhm A Anderer U Weigert M Sack U Automatic analysis of immunofluorescence patterns of HEp-2 cells Ann N Y Acad Sci 2007 1109 358 371 10.1196/annals.1398.042 17785325

[B20] Vincent L Soille P Watersheds in digital spaces: an efficient algorithm based on immersion simulations IEEE Trans Pattern Anal Mach Intell 1991 13 583 598 10.1109/34.87344

[B21] Cohen J A coefficient of agreement for nominal scales Educational and Psychological Measurement 1960 20 37 46 10.1177/001316446002000104

[B22] Tan EM Autoantibodies to nuclear antigens (ANA): their immunobiology and medicine Adv Immunol 1982 33 167 240 full_text 6182766 10.1016/s0065-2776(08)60836-6

[B23] Solomon DH Kavanaugh AJ Schur PH American College of Rheumatology Ad Hoc Committee on Immunologic Testing Guidelines Evidence-based guidelines for the use of immunologic tests: antinuclear antibody testing Arthritis Rheum 2002 47 434 444 10.1002/art.10561 12209492

[B24] Tan EM Cohen AS Fries JF Masi AT McShane DJ Rothfield NF Schaller JG Talal N Winchester RJ The revised criteria for the classification of systemic lupus erythematosus Arthritis Rheum 1982 25 1271 1277 10.1002/art.1780251101 7138600

[B25] Bayer PM Bauerfeind S Bienvenu J Fabien N Frei PC Gilburd B Heide KG Hoier-Madsen M Meroni PL Monier JC Monneret G Panzeri P Shoenfeld Y Spertini F Wiik A Multicenter evaluation study on a new HEp2 ANA screening enzyme immune assay J Autoimmun 1999 13 89 93 10.1006/jaut.1999.0298 10441172

[B26] Shovman O Gilburd B Barzilai O Shinar E Larida B Zandman-Goddard G Binder SR Shoenfeld Y Evaluation of the BioPlex 2200 ANA screen: analysis of 510 healthy subjects: incidence of natural/predictive autoantibodies Ann N Y Acad Sci 2005 1050 380 388 10.1196/annals.1313.120 16014555

[B27] Emlen W O'Neill L Clinical significance of antinuclear antibodies: comparison of detection with immunofluorescence and enzyme-linked immunosorbent assays Arthritis Rheum 1997 40 1612 1618 10.1002/art.1780400910 9324015

[B28] Kroshinsky D Stone JH Bloch DB Sepehr A Case records of the Massachusetts General Hospital. Case 5-2009. A 47-year-old woman with a rash and numbness and pain in the legs N Engl J Med 2009 360 711 720 10.1056/NEJMcpc0807822 19213685

[B29] Mahler M Ngo JT Schulte-Pelkum J Luettich T Fritzler MJ Limited reliability of the indirect immunofluorescence technique for the detection of anti-Rib-P antibodies Arthritis Res Ther 2008 10 R131 10.1186/ar2548 19000323 PMC2656233

[B30] Conrad K Ittenson A Reinhold D Fischer R Roggenbuck D Büttner T Bosselmann HP Steinbach J Schössler W High sensitive detection of double-stranded DNA autoantibodies by a modified *Crithidia luciliae *immunofluorescence test Ann N Y Acad Sci 2009 1173 180 185 10.1111/j.1749-6632.2009.04801.x 19758148

[B31] Nordal EB Songstad NT Berntson L Moen T Straume B Rygg M Biomarkers of chronic uveitis in juvenile idiopathic arthritis: predictive value of antihistone antibodies and antinuclear antibodies J Rheumatol 2009 36 1737 1743 10.3899/jrheum.081318 19567622

[B32] Rigon A Soda P Zennaro D Iannello G Afeltra A Indirect immunofluorescence in autoimmune diseases: assessment of digital images for diagnostic purpose Cytometry B Clin Cytom 2007 72 472 477 17549740 10.1002/cyto.b.20356

[B33] Soda P Early experiences in the staining pattern classification of HEp-2 slides Proceedings of the Twentieth IEEE International Symposium on Computer-Based Medical Systems 2007 Washington, DC: IEEE Computer Society 219 224 full_text

[B34] Hu Y Murphy RF Automated interpretation of subcellular patterns from immunofluorescence microscopy J Immunol Methods 2004 290 93 105 10.1016/j.jim.2004.04.011 15261574

[B35] Kang I Siperstein R Quan T Breitenstein ML Utility of age, gender, ANA titer and pattern as predictors of anti-ENA and -dsDNA antibodies Clin Rheumatol 2004 23 509 515 10.1007/s10067-004-0937-0 15801070

[B36] Reimer G Steen VD Penning CA Medsger TA Tan EM Correlates between autoantibodies to nucleolar antigens and clinical features in patients with systemic sclerosis (scleroderma) Arthritis Rheum 1988 31 525 532 10.1002/art.1780310409 2451921

[B37] Glory E Murphy RF Automated subcellular location determination and high throughput microscopy Dev Cell 2007 12 7 16 10.1016/j.devcel.2006.12.007 17199037

[B38] Hiemann R Hilger N Sack U Weigert M Objective quality evaluation of fluorescence images to optimize automatic image acquisition Cytometry A 2006 69 182 184 10.1002/cyto.a.20224 16496376

[B39] Perner P Perner H Müller B Mining knowledge for HEp-2 cell image classification Artif Intell Med 2002 26 161 173 10.1016/S0933-3657(02)00057-X 12234722

[B40] Soda P Iannello G A multi-expert system to classify fluorescent intensity in antinuclear autoantibodies testing Proceedings of the Nineteenth IEEE International Symposium on Computer-Based Medical Systems 2006 Washington, DC: IEEE ComputerSociety 219 224

[B41] Hollingsworth PN Dawkins RI Peter JB Precise quantitation of antinuclear antibodies on HEp-2 cells without the need for serial dilution Clin Diagn Lab Immunol 1996 3 374 377 8807199 10.1128/cdli.3.4.374-377.1996 PMC170353

